# Fatigue and Arousal Modulations Revealed by Saccade and Pupil Dynamics

**DOI:** 10.3390/ijerph19159234

**Published:** 2022-07-28

**Authors:** Jui-Tai Chen, Ying-Chun Kuo, Tzu-Yu Hsu, Chin-An Wang

**Affiliations:** 1 Department of Anesthesiology, School of Medicine, College of Medicine, Taipei Medical University, Taipei 110, Taiwan; cjuitai@tmu.edu.tw; 2 Department of Anesthesiology, Shuang Ho Hospital, Taipei Medical University, New Taipei City 235, Taiwan; 3 Institute of Cognitive Neuroscience, College of Health Science and Technology, National Central University, Taoyuan City 320, Taiwan; pupilncu@gmail.com; 4 Cognitive Intelligence and Precision Healthcare Research Center, National Central University, Taoyuan City 320, Taiwan; 5 Graduate Institute of Mind, Brain, and Consciousness (GIMBC), Taipei Medical University, Taipei 110, Taiwan; tzuyu.hsu@tmu.edu.tw; 6 Brain and Consciousness Research Center (BCRC), TMU-Shuang Ho Hospital, New Taipei City 235, Taiwan

**Keywords:** arousal, voluntary and reflexive saccades, saccade peak velocity, pupil light reflex, pupil dilation, superior colliculus, time-on-task, trial-to-trial

## Abstract

Saccadic eye movements are directed to the objects of interests and enable high-resolution visual images in the exploration of the visual world. There is a trial-to-trial variation in saccade dynamics even in a simple task, possibly attributed to arousal fluctuations. Previous studies have showed that an increase of fatigue level over time, also known as time-on-task, can be revealed by saccade peak velocity. In addition, pupil size, controlled by the autonomic nervous system, has long been used as an arousal index. However, limited research has been done with regards to the relation between pupil size and saccade behavior in the context of trial-to-trial variation. To investigate fatigue and arousal effects on saccadic and pupillary responses, we used bright and emotional stimuli to evoke pupillary responses in tasks requiring reactive and voluntary saccade generation. Decreased voluntary saccade peak velocities, reduced tonic pupil size and phasic pupillary responses were observed as time-on-task increased. Moreover, tonic pupil size affected saccade latency and dynamics, with steeper saccade main sequence slope as tonic pupil size increased. In summary, saccade dynamics and tonic pupil size were sensitive to fatigue and arousal level, together providing valuable information for the understanding of human behavior.

## 1. Introduction

Human performance is subject to a level of variability, and this trial-to-trial (or trial-by-trial) variability is associated with arousal fluctuations, known as a state of physiological activation through external or internal stimulation. A natural task to assess human performance is to study saccadic eye movements [1]. Saccades, the rapid eye movements that occur three to four times per second, are used to move the high acuity fovea in the exploration of the visual scene. There are two types of saccades: reactive (also known as reflexive) saccades are mainly driven by visual stimulation, whereas voluntary saccades require volitional control [2]. Saccade latency and dynamics are controlled by a network of brain areas, including the superior colliculus (SC) and frontal eye field (FEF), providing valuable insights into the understanding of sensory, cognitive and affective processing [3,4,5,6].

Human performance is under the influence of fatigue level [7,8]. Eye tracking has been used to develop an effective model for fatigue detection [9,10]. A body of research has shown that saccade metrics such as saccade peak velocity, amplitude, duration, and main sequence slope (peak velocity/amplitude) are sensitive to fatigue level, (e.g., [11,12,13,14,15]; review [16]). For example, Zargari et al. (2018) found an increase of fatigue level over time (referred to as time-on-task effects: time spent performing the task) correlating with reduced saccade peak velocities and increased saccade duration. While providing great insights into the relationship between fatigue and saccade dynamics, these investigations did not examine these relationships in different types of saccades in the context of trial-to-trial variation. It has yet to be determined which type of saccade is more sensitive to fatigue level.

Pupil size is controlled by the balance activity between the sympathetic and parasympathetic system [17,18]. The pupil transiently changes following external events (herein referred to as phasic pupillary responses). The pupil constricts in response to global luminance increases, known as the pupil light reflex, to regulate the amount of light projected onto the retina for optimized visual processing [19,20,21,22]. Additionally, pupil dilation is observed after the appearance of emotional stimuli [23,24,25,26,27,28,29]. More interestingly, the areas that are causally involved in the control of saccadic eye movements, including the SC and FEF, have recently been shown to also play a central role in the control of pupil size [30,31,32,33,34,35,36,37,38]. This raises an intriguing question: are phasic pupillary responses, similar to saccades, also modulated by fatigue level?

Pupil size has long been characterized as an index of arousal [39]. Baseline absolute pupil size (herein referred to as tonic pupil size) correlates with skin and heart rate responses on a trial-to-trial basis, e.g., [40]. Furthermore, pupil size tracks neural activity across various brain areas [32,41,42,43,44,45], including the locus coeruleus (LC), a structure causally involved in arousal regulation [41,46,47,48,49]. These results suggest that tonic pupil size is an effective indicator of the general arousal level. However, there is limited research that focuses on examining the relation of tonic pupil size to stimulus-evoked saccade and pupillary responses on a trial-to-trial basis.

To investigate fatigue (i.e., time-on-task) and arousal (i.e., tonic pupil size) effects on saccade and pupil behavior, we used bright and emotional stimuli to respectively evoke pupil constriction and dilation responses in tasks involving reactive and voluntary saccades. We hypothesize that fatigue and arousal effects should be revealed by saccade peak velocity and phasic pupillary responses on a trial-to-trial basis because of the involvement of overlapping neural circuitry via the SC.

## 2. Methods and Materials

*Experimental setup.* All experimental procedures were reviewed and approved by the Institutional Review Board of the Taipei Medical University (N201903086; N201909070), Taiwan, and were in accordance with the Declaration of Helsinki [50]. Twenty-eight (8 males, mean age: 28.1, SD: 3.8 years) and twenty-four (11 males, mean age: 25.7, SD: 4 years) healthy participants from Taipei Medical University were recruited for Experiments 1 and 2, respectively. Participants had normal or corrected-to-normal vision and were naïve regarding the purpose of the experiment. Participants provided informed consent and were compensated financially for their participation.

*Recording and Apparatus.* Participants were seated in a dark room. As described previously [51], eye position and pupil size were measured with a video-based eye tracker (Eyelink-1000 plus binocular-arm, SR Research, Osgoode, ON, Canada) at a rate of 500 Hz with binocular recording (left pupil was used), and stimulus presentation and data acquisition were controlled by the Eyelink Experiment Builder. Stimuli were presented on an LCD monitor at a screen resolution of 1920 × 1080 pixels (60 Hz refresh rate), subtending a viewing angle of 58° × 32°, with the distance from the eyes to the monitor set at 60 cm.

**Figure 1:**
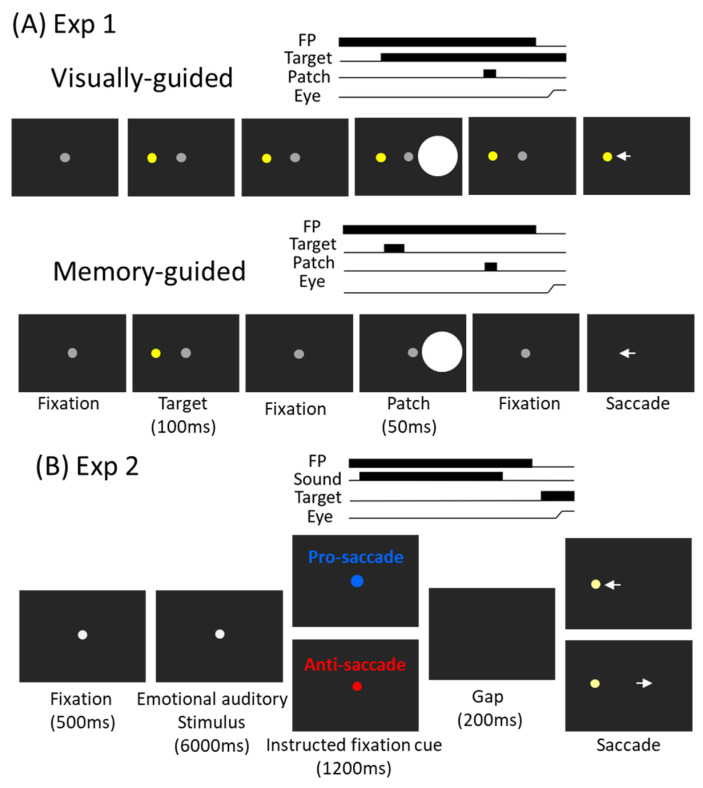
(**A**) **Experiment 1 paradigm**. Each trial started with a central fixation point on a black background. After a delay, there was a presentation of a target stimulus, and after a random delay the central fixation point disappeared and participants were required to move their eyes to the target. During the delay period, a bright circular patch stimulus was presented briefly (50 ms), with the patch being spatially aligned with the target location or the opposite location of the target. The memory-guided task was similar to the visually guided task, except the target stimulus was only presented briefly (100 ms). Note that the figure only shows one condition for illustration of the paradigm. (**B**) **Experiment 2 paradigm**. Each trial began with a central fixation point on a background. After a delay, an emotional auditory stimulus was presented for 6 s, followed by the instructed colored fixation cue (1200 ms) for the pro- or anti-saccade condition. A blank screen was presented for 200 ms (gap) before target stimulus presentation, and participants were required to move their eyes to the target in the pro-saccade condition or look away to the opposite location in the anti-saccade condition, after disappearance of the central fixation point.

*Experiment 1: Visually guided and memory-guided saccade task* (Figure 1A). We used visually guided and memory-guided saccade tasks because these saccades are well studied, also enabling saccades guided by visual signals (visually guided) to be distinguished from others [52,53,54,55,56]. Second, a bright stimulus was presented in this task, allowing us to also examine fatigue and arousal effects on phasic pupil constriction responses. The visually guided and memory-guided patch paradigm [57] was modified so that all task conditions were interleaved to investigate trial-to-trial variability effects between the two saccade task conditions. Specifically, participants were seated in a dark room, and the experiment had 2 tasks (visually and memory-guided) that were intermixed within a block of 335 trials lasting approximately 45 min. In the visually guided task, each trial began with the appearance of a central fixation point (FP) (0.5° diameter; ~10 cd/m^2^) on a black background (~0.01 cd/m^2^). After a period (800–900 ms), a peripheral colored target (0.5° diameter; ~45 cd/m^2^; referred to as the target stimulus) appeared to the right or left (radial angle: 0 or 180°) at an eccentricity of 7–9° visual angle from the central FP. After a variable delay (500–800 ms), a bright circular patch was displayed briefly for 50 ms (6° in diameter, ~50 cd/m^2^, referred to as the patch stimulus). After another variable delay (1200–1350 ms), the FP was removed, and participants were required to generate a saccade toward the target. The patch stimulus location was spatially aligned with the target location or presented in the mirror location of the target stimulus with the target location (each condition had ~40% of trials). In catch trials (~17% of trials), no patch stimulus was presented, such that after a variable delay (500–800 ms) following the target onset, the FP was removed, and participants were required to generate a saccade toward the target. In the memory-guided task, the configuration was identical to the visually guided configuration, except that the target was only presented for 100 ms. Again, task condition (visually guided or memory-guided), target location (left and right) and patch location (left and right) were randomly interleaved. This experiment was part of a project involving continuous theta-burst stimulation using transcranial magnetic stimulation. Thus, the participants came in twice (one week apart) for the same experiment with vertex or frontal eye field stimulation. Here, we only focus on the data with vertex stimulation.

*Experiment 2: Emotional sounds with pro- and anti-saccade task* (Figure 1B). We used this task because we intended to investigate fatigue and arousal effects on reactive and voluntary saccades, as pro- and anti-saccades are representative of those types of saccades [4,5]. Moreover, we intended to examine fatigue and arousal effects on phasic pupil dilation responses, and this task used emotional sounds to induce pupil dilation. Aspects of Experiment 2 have been published previously [29], and the method is described in detail. Briefly, the experiment consisted of 156 trials lasting approximately 80 min. Each trial began with the appearance of a central fixation point (FP) (0.5° diameter, grey color, 20 cd/m^2^) on a background in a dark room. After 500 ms of central fixation, an emotional auditory stimulus from the International Affective Digital Sounds (IADS) [58] was presented through headphones with a central FP for 6000 ms. Afterward, the color of the FP changed in accordance with the saccade task condition (the luminance level of the two FP colors were matched, 20 cd/m^2^). On pro-saccade trials, participants were instructed to look towards the peripheral target stimulus as soon as it appeared. On anti-saccade trials, participants were instructed to look in the opposite direction of the target stimulus as soon as it appeared. After another 1200 ms of central fixation, the FP disappeared for 200 ms (gap) before the peripheral target stimulus appeared (0.5° diameter, 270 cd/m^2^) to the left or right of the FP (8° eccentricity on the horizontal axis). The experiment with a dark background (2 cd/m^2^) was used because its fatigue effects associated with microsaccades are more pronounced [59]. Notably, pro- and anti-saccade are respectively considered as reactive and voluntary saccades [4,5], because unlike the automatic visuomotor response required in the pro-saccade condition, to complete an anti-saccade, subjects must suppress the automatic saccade and generate a voluntary response in the opposite direction of the stimulus.

*Data analysis.* Saccade reaction time (SRT) was defined as the time from fixation disappearance to the first saccade away from fixation (eye velocity exceeded 30°/s) with an amplitude greater than 3°. Trials were scored as correct if the first saccade after stimulus appearance was in the correct direction (toward the target). The saccades with SRTs of <90 ms were classified as anticipatory [60], and failure to initiate a saccade within 1200 ms after the disappearance of FP was considered as lack of attention; thus, these trials were excluded from analysis. To maintain accurate measurement of pupil size around the patch presentation period before saccade initiation, trials with an eye position deviation of more than 2° from the central FP or with detected saccades (>2° amplitude) during the period from 500 ms before to 1200 ms after patch onset were excluded from analysis. When blinks were detected, following the literature, pre- and post-blink pupil values were used to perform a linear interpolation to replace pupil values during the blink period [61,62]. Note that outlier values beyond 1.5 times the interquartile range (the difference between upper and lower quartiles) below the lower quartile or above the upper quartile were also excluded from analysis. Saccade metrics including saccade amplitude (saccade size in degree), saccade peak velocity (deg/s), and main sequence slope (peak velocity/amplitude) were also analyzed to understand the influence of fatigue and arousal on saccade dynamics.

We used a baseline-correction procedure for each trial [63,64], and pupil values were subtracted from this baseline value in each trial. In Experiment 1, a baseline value was determined by averaging pupil size from 100 ms before to the patch stimulus onset, and absolute pupil size in this epoch was used to index tonic pupil size. To capture the peak pupil constriction response after the patch presentation, an epoch of 600 to 700 ms after the patch presentation was used because the time to peak constriction was ~650 ms. In Experiment 2, for emotional sound pupil analysis, a baseline value was determined by averaging pupil size from auditory stimulus onset to 100 ms after its appearance, and absolute pupil size in this epoch was used to index tonic pupil size. To capture phasic pupil dilation evoked by emotional sounds, following our previous results [29], an epoch of 2000 to 6000 ms after the auditory stimulus presentation was used. To illustrate time-on-task effects, we divided the total number of trials into three time-on-task categories with an approximately equal number of trials in each. For a given subject, category Begin has a trial number less than 1/3 of the total number of trials. Mid has a trial number between 1/3 and 2/3 of the total number of trials, and End contains a trial number exceeding 2/3 of the total number of trials. Similarly, to illustrate tonic pupil size effects, we ranked trials according to the tonic pupil size and then divided them into three tonic pupil size categories, with an approximately equal number of trials in each. Small contains tonic pupil size trials less than 1/3 of trials, Mid has tonic pupil size between 1/3 and 2/3 of trials, and Large has tonic pupil size exceeding 2/3 of trials. A two-way (or one-way) repeated-measure ANOVA was used to examine effects of saccade type (visually and memory-guided saccade or pro- and anti-saccade) and trial number (or tonic pupil size) on the saccade and pupillary response. More importantly, an independent two-tailed student *t*-test was performed to inform whether trial-to-trial correlation at the population level (the correlation coefficients of all participants) was significant. Effect sizes (partial eta squared or Cohen’s d), where appropriate, were also reported. Statistical tests were performed using JASP Team (2019) [65] and MATLAB (The MathWorks Inc., Natrick, MA, USA).

## 3. Results

### 3.1. Exp 1: Time-On-Task Effects on Saccade Latency and Metrics in the Visually and Memory-Guided Saccade Task

To investigate the fatigue effects on saccade latency and metrics, we correlated trial number with saccade latency and metrics under the assumption that fatigue level increases as time spent performing the task increases (time-on-task effects). Moreover, as mentioned, visually-guided and memory-guided saccade tasks were used to differentiate saccades guided by visual signals from others [52,53,54,55,56]. Participants performed the tasks accurately with correct responses made for 96 ± 1% (mean ± s.e.m.) of trials in the visually guided saccade task and 95 ± 1% of trials in the memory-guided saccade task, and correct trials were included for the following analyses. Mean saccadic reaction times (SRT) were 214 ± 7 ms in the visually guided task and 213 ± 6 ms in the memory-guided task.

**Figure 2:**
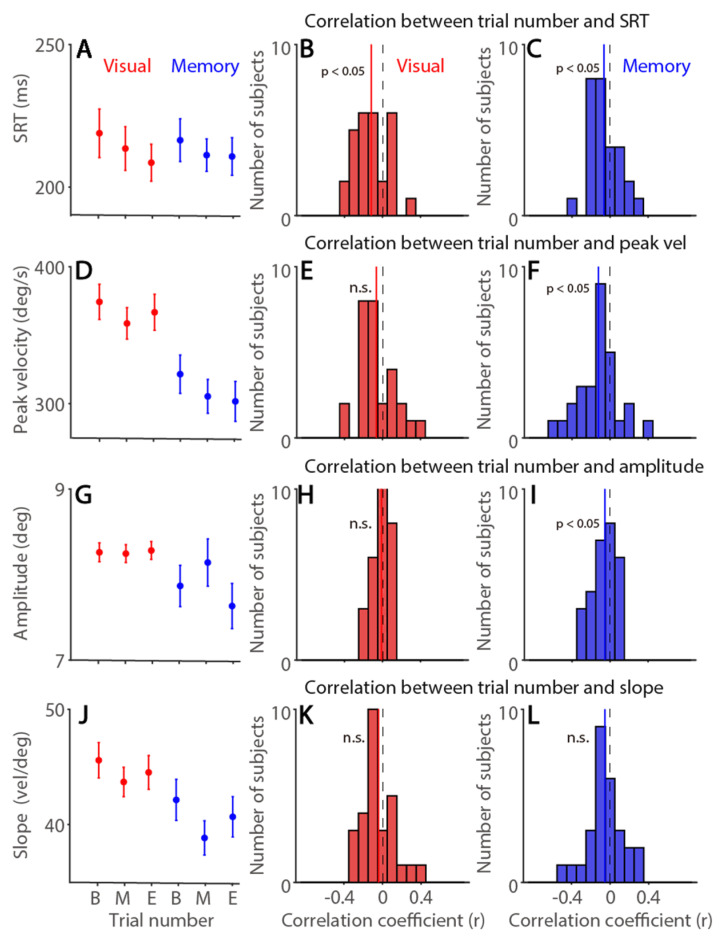
**Time-on-task effects on visually and memory-guided saccade latency and metrics.** (**A**) Saccade reaction times (SRTs) at different trial number conditions. (**B**,**C**) Distribution of correlation coefficients for the relationship between trial number and SRT in visually guided (**B**) and memory-guided (**C**) saccades for all subjects (*n* = 28). (**D**) Saccade peak velocity at different trial number conditions. (**E**,**F**) Distribution of correlation coefficients for the relationship between trial number and saccade peak velocity in visually guided (**E**) and memory-guided (**F**) saccades for all subjects. (**G**) Saccade amplitude at different trial number conditions. (**H**,**I**) Distribution of correlation coefficients for the relationship between trial number and saccade amplitude in visually guided (**H**) and memory-guided (**I**) saccades for all subjects. (**J**) Main sequence slope (peak velocity/amplitude) at different trial number conditions. (**K**,**L**) Distribution of correlation coefficients for the relationship between trial number and slope in visually guided (**K**) and memory-guided (**L**) saccades for all subjects. The error bars represent ± standard error range (across participants). The vertical black dashed line represents a zero value of the correlation coefficient (r = 0), and the vertical colored line represents the mean value of the correlation coefficient. Visual: visually guided saccade task. Memory: memory-guided saccade task. B: begin, M: mid, E: end. SRT: saccade reaction times. n.s.: not significant.

To examine the fatigue effect on visually and memory-guided saccades, we first divided trials into three time-on-task categories, Begin, Mid, End (see Methods and Materials). As shown in Figure 2A, SRTs were significantly modulated by time-on-task (F(2,54) = 5.285, *p* = 0.008, η*_p_*^2^ = 0.164), that is, faster SRTs were obtained as trial number increased. This could be due to task-related familiarity factors increased over time. Other effects were not significant (ps > 0.5). To investigate trial-to-trial correlation between trial number and SRTs across all participants, we performed correlational analysis in the visually- and memory-guided task separately (Table 1 for summary). In the visually guided task, as displayed in Figure 2B (histograms of the correlation coefficients), negative correlation was obtained (*t*(27) = 3.451, *p* = 0.002, d = 0.652), and similar effects were observed in the memory-guided task (Figure 2C, *t*(27) = 2.146, *p* = 0.041, d = 0.405).

To further investigate whether fatigue also affected saccade dynamics, we examined saccade peak velocity, amplitude, and main sequence slope (peak velocity/amplitude). Saccade peak velocities were significantly modulated by time-on-task, with decreased peak velocities as trial number increased (Figure 2D, F(2,54) = 5.688, *p* = 0.006, η*_p_*^2^ = 0.174), and higher saccade peak velocities obtained in visually guided, compared to memory-guided, saccades (F(1,27) = 121.790, *p* < 0.001, η*_p_*^2^ = 0.819), and interaction was significant (F(2,54) = 6.398, *p* = 0.003, η*_p_*^2^ = 0.192). Moreover, trial-to-trial correlational analysis showed significant negative correlations between trial number and saccade peak velocity in the memory-guided task (Table 1, Figure 2F, *t*(27) = 3.003, *p* < 0.001, d = 0.568) but not in the visually-guided task (Figure 2E, *t*(27) = 1.851, *p* = 0.075, d = 0.350). These results are generally in line with previous research [11,12,13,15] showing that saccade peak velocity is sensitive to fatigue level and was thus decreased as time spent on the task increased. Saccade amplitude was modulated by time-on-task (Figure 2G, F(2,54) = 12.652, *p* < 0.001, η*_p_*^2^ = 0.319), and effects of task and interaction were significant (task: F(1,27) = 4.454, *p* = 0.044, η*_p_*^2^ = 0.142; interaction: F(2,54) = 26.534, *p* < 0.001, η*_p_*^2^ = 0.496). However, trial-to-trial correlation was only significant in the memory-guided task, with smaller saccade amplitude as time spent on the task increased (visual: Figure 2H, *t*(27) = 0.587, *p* = 0.562, d = 0.111; memory: Figure 2I, *t*(27) = 2.322, *p* = 0.028, d = 0.439). As shown in Figure 2J, the main sequence slope was modulated by trial number (F(2,54) = 10.009, *p* < 0.001, η*_p_*^2^ = 0.270), and effects of task and interaction were significant (task: F(1,27) = 19.477, *p* < 0.001, η*_p_*^2^ = 0.419; interaction: F(2,54) = 3.877, *p* = 0.027, η*_p_*^2^ = 0.126). However, these effects were not significant on trial-to-trial analysis in both tasks (visual: Figure 2K, *t*(27) = 1.316, *p* = 0.199, d = 0.249; memory: Figure 2L, *t*(27) = 1.521, *p* = 0.140, d = 0.287). Overall, time-on-task effects were particularly pronounced in memory-guided saccades.

### 3.2. Time-On-Task Effects on Tonic Pupil Size and Phasic Pupil Light Reflex Responses

**Figure 3:**
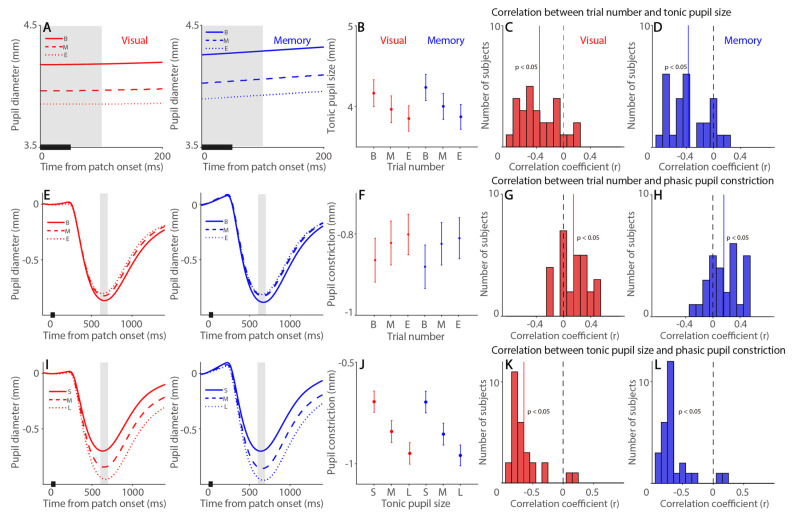
**Time-on-task effects on tonic pupil size and phasic pupil constriction.** (**A**) Dynamics of absolute pupil diameter following the presentation of bright patch stimuli. (**B**) Tonic pupil size (0 to 100 ms after patch onset) in different trial number conditions in the visually guided and memory-guided saccade task. (**C**,**D**) Distribution of correlation coefficients for the relationship between trial number and tonic pupil size in the visually guided (**C**) and memory-guided (**D**) saccade task for all subjects (*n* = 28). (**E**) Changes in pupil diameter following the presentation of bright patch stimuli. (**F**) Pupil constriction size (600 to 700 ms after patch onset) in different trial number conditions on the visually guided and memory-guided saccade task. (**G**,**H**) Distribution of correlation coefficients for the relationship between trial number and phasic pupil constriction size in the visually guided (**G**) and memory-guided (**H**) saccade task for all subjects. (**I**) Changes in pupil diameter following the presentation of bright patch stimuli. (**J**) Pupil constriction size (600 to 700 ms after patch onset) in different tonic pupil size conditions in the visually guided and memory-guided saccade task. (**K**,**L**) Distribution of correlation coefficients for the relationship between tonic pupil size and phasic pupil constriction size in the visually guided (**K**) and memory-guided (**L**) saccade task for all subjects. In (**A**,**E**,**I**), the black bar on X-axis indicates the time line of patch presentation. The analyzed epochs are shaded in gray. In (**B**,**F**,**J**), the error bars represent ± standard error range (across participants). In (**C**,**D**,**G**,**H**,**K**,**L**), the vertical black dashed line represents a zero value of the correlation coefficient (r = 0), and the vertical colored line represents the mean value of the correlation coefficient. Visual: visually guided saccade task. Memory: memory-guided saccade task. B: begin, M: mid, E: end. S: small, L: large.

Pupil size, as an index of arousal level, is associated with peripheral indices [40], and fatigue is one of the key arousal factors that influences task performance [9,10,13]. Moreover, because tonic pupil size and phasic pupillary responses are thought to reveal different neural processes [41,66,67], we examined time-on-task effects on tonic pupil size and phasic pupillary responses separately. To investigate the link between fatigue level and baseline tonic pupil size (100 me before to bright stimulus onset), we first examined the relation between trial number and tonic pupil size. As shown in Figure 3A, tonic pupil size was significantly modulated by time-on-task (Figure 3B, F(2,54) = 21.700, *p* < 0.001, η*_p_*^2^ = 0.446), and effects of task and interaction were also significant (task: F(1,27) = 16.755, *p* < 0.001, η*_p_*^2^ = 0.446; interaction: F(2,54) = 3.306, *p* = 0.044, η*_p_*^2^ = 0.109). Importantly, trial-to-trial correlational analysis further showed significant negative correlations between trial number and tonic pupil size in both tasks (visual: Figure 3C, *t*(27) = 6.596, *p* < 0.001, d = 1.247; memory: Figure 3D, *t*(27) = 6.785, *p* < 0.001, d = 1.282), with smaller pupil sizes as trial number increased. To examine whether phasic pupillary responses, i.e., the pupil light reflex, were modulated by time-on-task effects, we examined the relation between trial number with phasic pupillary responses. As displayed in Figure 3E, the pupil constricted after the presentation of a bright stimulus in both tasks, and the size of max constriction was modulated by time-on-task (Figure 3F, F(2,54) = 12.557, *p* < 0.001, η*_p_*^2^ = 0.317), and effects of task and interaction were not significant (task: F(1,27) = 2.261, *p* = 0.144, η*_p_*^2^ = 0.077; interaction: F(2,54) = 1.069, *p* = 0.350, η*_p_*^2^ = 0.038). Trial-to-trial correlational analysis further showed significant positive correlations between trial number and phasic pupillary responses in both tasks (visual: Figure 3G, *t*(27) = 3.959, *p* < 0.001, d = 0.748; memory: Figure 3H, *t*(27) = 3.649, *p* < 0.001, d = 0.69), with smaller constriction sizes as trial number increased. In summary, smaller tonic pupil size and phasic pupillary responses were observed as time-on-task increased.

To directly examine whether tonic pupil size, as an index of arousal, affected phasic pupillary responses, we first ranked trials according to the tonic pupil size into three time-on-task categories: Small, Mid and Large (see Methods and Materials). As shown in Figure 3I, phasic pupil constriction responses were modulated by tonic pupil size, with smaller pupil constriction (smaller negative values) in trials with smaller tonic pupil size (Figure 3J, F(2,54) = 116.199, *p* < 0.001, η*_p_*^2^ = 0.811). Effects of task and interaction were not significant (task: F(1,27) = 1.5, *p* = 0.231, η*_p_*^2^ = 0.053; interaction: F(2,54) = 0.671, *p* = 0.515, η*_p_*^2^ = 0.024). As shown in Figure 3K,L for trial-to-trial correlational analysis (Table 1), smaller pupil constriction was observed when tonic pupil sizes were smaller (visual: *t*(27) = 12.759, *p* < 0.001, d = 2.411; memory: *t*(27) = 12.8, *p* < 0.001, d = 2.419). Moreover, because phasic pupil constriction also correlated with trial number, we computed the partial correlation coefficient to control the effect of trial number. As shown in Supplementary Figure S1, tonic pupil size still significantly correlated with phasic pupil constriction size (Supplementary Table S1 for summary). Notably, because there is more room for the pupil to constrict when tonic pupil size is larger, this correlation between tonic pupil size and phasic pupillary constriction responses can be explained by the law of initial values [68].

### 3.3. Tonic Pupil Size Effects on Saccade Latency and Metrics in the Visually and Memory-Guided Saccade Task

**Figure 4:**
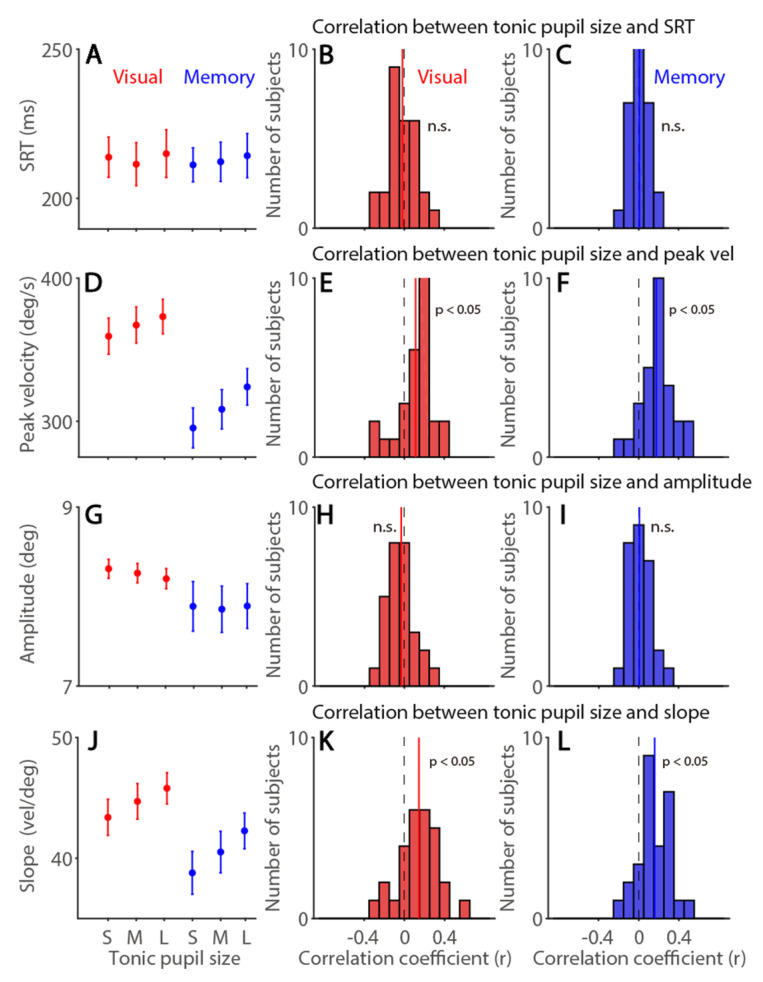
**Tonic pupil size effects on visually and memory-guided saccade latency and metrics.** (**A**) SRTs at different tonic pupil size conditions. (**B**,**C**) Distribution of correlation coefficients for the relationship between tonic pupil size and SRT in visually guided (**B**) and memory-guided (**C**) saccades for all subjects (*n* = 28). (**D**) Saccade peak velocity at different tonic pupil size conditions. (**E**,**F**) Distribution of correlation coefficients for the relationship between tonic pupil size and saccade peak velocity in visually guided (**E**) and memory-guided (**F**) saccades for all subjects. (**G**) Saccade amplitude at different tonic pupil size conditions. (**H**,**I**) Distribution of correlation coefficients for the relationship between tonic pupil size and saccade amplitude in visually guided (**H**) and memory-guided (**I**) saccades for all subjects. (**J**) Main sequence slope (peak velocity/amplitude) at different tonic pupil size conditions. (**K**,**L**) Distribution of correlation coefficients for the relationship between tonic pupil size and slope in visually guided (**K**) and memory-guided (**L**) saccades for all subjects. The error bars represent ± standard error range (across participants). The vertical black dashed line represents a zero value of the correlation coefficient (r = 0), and the vertical colored line represents the mean value of the correlation coefficient. Visual: visually guided saccade task. Memory: memory-guided saccade task. S: small, M: mid, L: large. SRT: saccade reaction times. n.s.: not significant.

Arousal is a complicated concept consisting of different components, and tonic pupil size is linked to autonomic indices, e.g., [19], as an effective index for the general arousal level [69]. To investigate tonic pupil size effects on saccade latency and metrics, we correlated tonic pupil size with saccade latency and metrics. As shown in Figure 4A, SRTs were not modulated by tonic pupil size or by other factors (tonic pupil size: F(2,54) = 1.444, *p* = 0.245, η*_p_*^2^ = 0.051; task: F(1,27) = 0.045, *p* = 0.834, η*_p_*^2^ = 0.002; interaction: F(2,54) = 0.415, *p* = 0.663, η*_p_*^2^ = 0.015). Trial-to-trial correlations between tonic pupil size and SRTs were not significant in both tasks (Table 1, Figure 4B: visual: *t*(27) = 0.678, *p* = 0.504, d = 0.128; Figure 4C: memory: *t*(27) = 0.383, *p* = 0.705, d = 0.072). In contrast, saccade peak velocities were significantly modulated by tonic pupil size (Figure 4D, F(2,54) = 22.403, *p* < 0.001, η*_p_*^2^ = 0.453), and effects of task and interaction were also significant (task: F(1,27) = 121.414, *p* < 0.001, η*_p_*^2^ = 0.818; interaction: F(2,54) = 5.229, *p* = 0.008, η*_p_*^2^ = 0.162). Trial-to-trial correlational analysis further showed significant positive correlations between tonic pupil size and saccade peak velocities in both tasks (Figure 4E: visual: *t*(27) = 3.505, *p* = 0.002, d = 0.662; Figure 4F: memory: *t*(27) = 5.920, *p* < 0.001, d = 1.119), with larger tonic pupil size correlating with higher peak velocities. Saccade amplitude was not modulated by tonic pupil size (Figure 4G, time: F(2,54) = 0.533, *p* = 0.59, η*_p_*^2^ = 0.019; task: F(1,27) = 4.233, *p* = 0.049, η*_p_*^2^ = 0.138; interaction: F(2,54) = 1.01, *p* = 0.371, η*_p_*^2^ = 0.036). No trial-to-trial correlations between tonic pupil size and saccade amplitude were observed (Figure 4H: visual: *t*(27) = 1.266, *p* = 0.216, d = 0.239; Figure 4I: memory: *t*(27) = 0.392, *p* = 0.745, d = 0.062). Main sequence slope was also modulated by tonic pupil size (Figure 4J, F(2,54) = 21.595, *p* < 0.001, η*_p_*^2^ = 0.444), and steeper slope was obtained in visually guided than memory-guided saccades (task: F(1,27) = 19.877, *p* < 0.001, η*_p_*^2^ = 0.424; interaction: F(2,54) = 1.382, *p* = 0.26, η*_p_*^2^ = 0.049). Trial-to-trial positive correlations between tonic pupil size and main sequence slope were observed in both tasks, with larger tonic pupil size correlating with steeper slope (Figure 4K: visual: *t*(27) = 3.954, *p* < 0.001, d = 0.747; Figure 4L: memory: *t*(27) = 5.414, *p* < 0.001, d = 1.023). Furthermore, because these saccade responses also correlated with trial number, we computed the partial correlation coefficient to control the effect of trial number (Supplementary Table S1 for summary). As shown in Supplementary Figure S2, the same patterns of the results were obtained in correlational analyses. Importantly, these results suggest that tonic pupil size, in addition to time-on-task, uniquely accounted for some fluctuations of saccade dynamics on a trial-to-trial basis. In summary, larger tonic pupil size correlated with higher saccade peak velocities and steeper main sequence slope.

### 3.4. Exp 2: Time-On-Task Effects on Tonic Pupil Size and Phasic Pupil Dilation in the Emotion Sound Task

In addition to pupil light reflex induced by global luminance increases [17,18], pupil dilation is evoked by emotional arousal [23,24,25,26,27,28,29]. As demonstrated in Experiment 1, pupil constriction responses were influenced by time-on-task, with smaller pupil constriction as time-on-task increased, and the size of evoked constriction also scaled with tonic pupil size. However, these observed effects are yet to be extended to pupil dilation responses. Moreover, the correlation between tonic pupil size and phasic pupil constriction size could be explained by the law of initial values [68], as there is more room for the pupil to constrict when tonic pupil size is larger. To examine the fatigue and tonic pupil size effects on pupil dilation responses and the reliability of these effects on saccade latency and dynamics, we analyzed an experiment that used emotional sounds to induce pupil dilation in an interleaved pro- and anti-saccade task.

### 3.5. Time-On-Task Effects on Tonic Pupil Size and Phasic Pupil Dilation Responses

**Figure 5:**
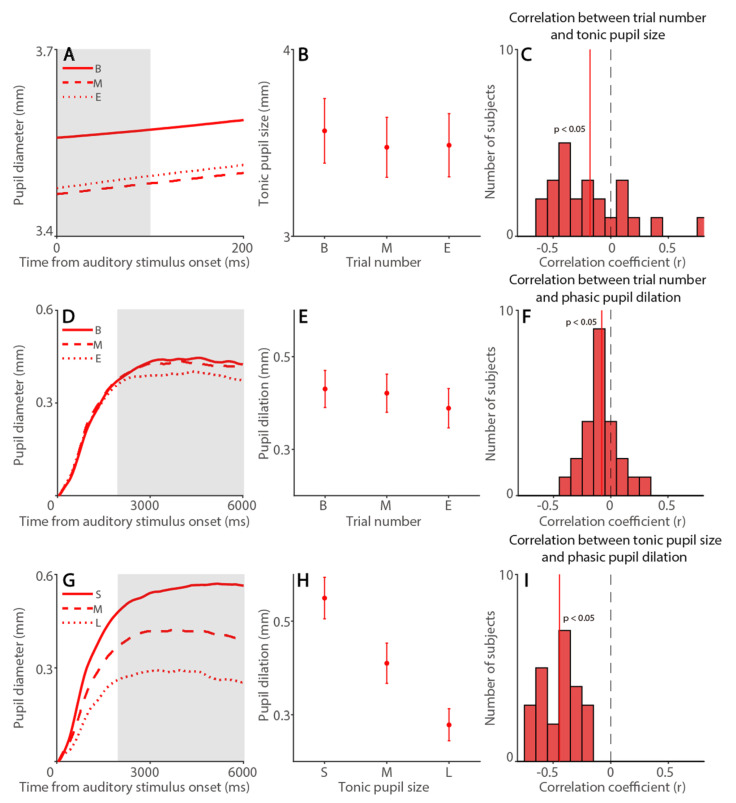
**Tonic pupil size effects on tonic pupil size and phasic pupil dilation.** (**A**) Dynamics of absolute pupil diameter following the presentation of emotional auditory stimuli. (**B**) Tonic pupil size (0 to 100 ms after emotional auditory stimulus onset) in different trial number conditions. (**C**) Distribution of correlation coefficients for the relationship between trial number and tonic pupil size for all subjects (*n* = 24). (**D**) Changes in pupil diameter following the presentation of bright patch stimuli. (**E**) Pupil dilation size (2000 to 6000 ms after emotional auditory stimulus onset) in different trial number conditions. (**F**) Distribution of correlation coefficients for the relationship between trial number and phasic pupil dilation size for all subjects. (**G**) Changes in pupil diameter following the presentation of emotional auditory stimuli. (**H**) Pupil dilation size (2000 to 6000 ms after patch onset) in different tonic pupil size conditions. (**I**) Distribution of correlation coefficients for the relationship between tonic pupil size and phasic pupil dilation size for all subjects. In (**A**,**D**,**G**), the analyzed epochs are shaded in gray. In (**B**,**E**,**H**), the error bars represent ± standard error range (across participants). In (**C**,**F**,**I**), the vertical black dashed line represents a zero value of the correlation coefficient (r = 0), and the vertical colored line represents the mean value of the correlation coefficient. B: begin, M: mid, E: end. S: small, L: large.

To examine the fatigue effect on tonic pupil size and phasic pupil dilation, as described previously, we followed the same procedure to divide trials into three time-on-task categories (Begin, Mid, End). As shown in Figure 5A, tonic pupil size (mean pupil size: 0–100 ms after emotional sound onset) was as a function of time-on-task conditions, though the main effect was not significant (Figure 5B, F(2,46) = 1.215, *p* = 0.306, η*_p_*^2^ = 0.05). Moreover, consistent with the results in Experiment 1, trial-to-trial correlational analysis between trial number and tonic pupil size showed significant negative correlations (Figure 5C, *t*(27) = 2.574, *p* = 0.017, d = 0.525), with decreased tonic pupil size as time-on-task increased (see Table 2 for summary). Baseline-corrected pupil dynamics following emotional auditory stimulus are shown in Figure 5D, with larger pupil dilation in the beginning of the trials (Figure 5E), though the effect was not significant (F(2,46) = 2.648, *p* = 0.082, η*_p_*^2^ = 0.103). Trial-to-trial correlation at the population level was significant (Figure 5F, *t*(27) = 2.487, *p* = 0.021, d = 0.508), showing reduced pupil dilation as time-on-task increased. These results were consistent with Experiment 1, that is, pupil constriction responses reduced as time spent on task increased. We followed the same procedure to divide trials into three tonic pupil size categories (Small, Mid, Large). Interestingly, in contrast to pupil constriction responses, pupil dilation responses were larger with smaller tonic pupil size (Figure 5G,H, F(2,46) = 92.649, *p* < 0.001, η*_p_*^2^ = 0.801). Significant correlations between tonic pupil size and pupil dilation magnitude were observed (Figure 5I, *t*(27) = 14.611, *p* < 0.001, d = 2.982), with smaller tonic pupil size correlating with larger pupil dilation. Again, we computed the partial correlation coefficient to control the effect of trial number. As shown in Supplementary Figure S3, tonic pupil size significantly correlated with phasic pupil dilation magnitude (Supplementary Table S2 for summary), suggesting that phasic pupillary responses were under the law of initial values [68].

### 3.6. Time-On-Task Effects on Saccade Latency and Metrics in the Pro- and Anti-Saccade Task

**Figure 6:**
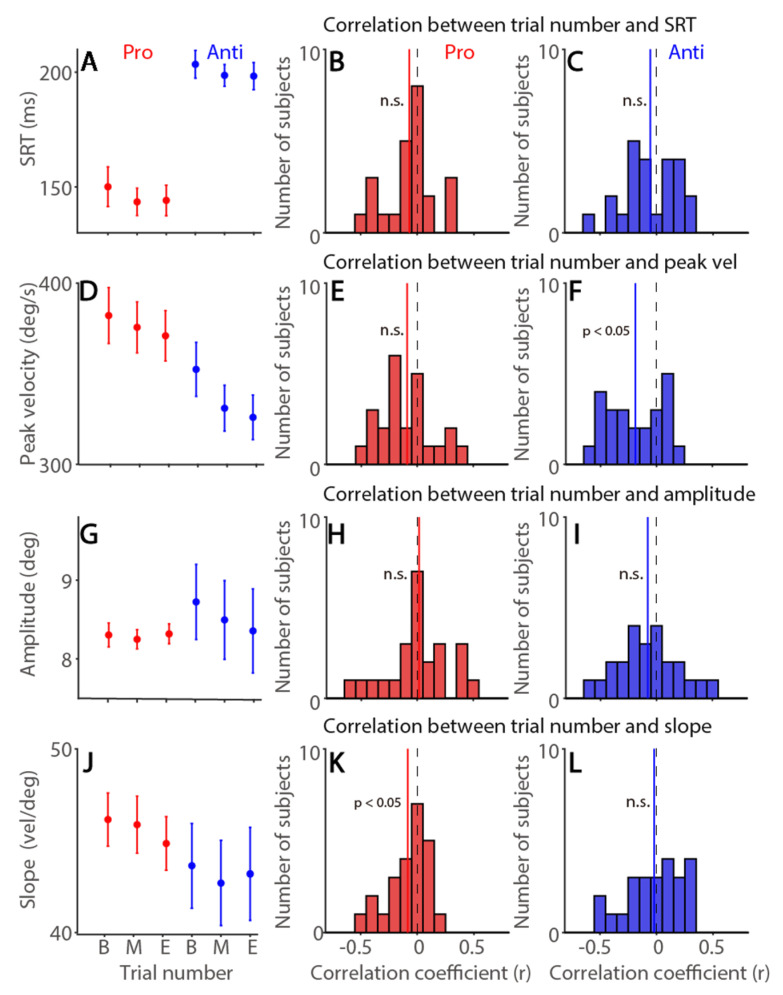
**Time-on-task effects on saccade latency and metrics in the pro- and anti-saccade task.** (**A**) Saccade reaction times (SRTs) at different trial number conditions. (**B**,**C**) Distribution of correlation coefficients for the relationship between trial number and SRT in the pro-saccade (**B**) and anti-saccade (**C**) task for all subjects (*n* = 24). (**D**) Saccade peak velocity at different trial number conditions. (**E**,**F**) Distribution of correlation coefficients for the relationship between trial number and saccade peak velocity in the pro-saccade (**E**) and anti-saccade (**F**) task for all subjects. (**G**) Saccade amplitude at different trial number conditions. (**H**,**I**) Distribution of correlation coefficients for the relationship between trial number and saccade amplitude in the pro-saccade (**H**) and anti-saccade (**I**) task for all subjects. (**J**) Main sequence slope (peak velocity/amplitude) at different trial number conditions. (**K**,**L**) Distribution of correlation coefficients for the relationship between trial number and slope in the pro-saccade (**K**) and anti-saccade (**L**) task for all subjects. The error bars represent ± standard error range (across participants). The vertical black dashed line represents a zero value of the correlation coefficient (r = 0), and the vertical colored line represents the mean value of the correlation coefficient. Visual: visually guided saccade task. Memory: memory-guided saccade task. B: begin, M: mid, E: end. SRT: saccade reaction times. n.s.: not significant.

To examine the fatigue effect on saccade latency and dynamics in the interleaved pro- and anti-saccade task (commonly referred to as reactive and voluntary saccades, respectively), correct trials were included for following analyses. As shown in Figure 6A, SRTs were not modulated by time-on-task (time: F(2,46) = 1.515, *p* = 0.231, η*_p_*^2^ = 0.062; task: F(1,23) = 84.419, *p* < 0.001, η*_p_*^2^ = 0.786; interaction: F(2,46) = 0.044, *p* = 0.957, η*_p_*^2^ = 0.002). No trial-to-trial correlations were observed in both tasks (Figure 6B: pro: mean correlation coefficient: −0.07, *t*(23) = 1.536, *p* = 0.138, d = 0.314; Figure 6C: anti: mean correlation coefficient: −0.05, *t*(23) = 1.142, *p* = 0.265, d = 0.233). In contrast, saccade peak velocities were under time-on-task effects (6D, F(2,46) = 9.874, *p* < 0.001, η*_p_*^2^ = 0.3), and effects of task and interaction were also significant (task: F(1,23) = 30.441, *p* < 0.001, η*_p_*^2^ = 0.57; interaction: F(2,46) = 4.153, *p* = 0.022, η*_p_*^2^ = 0.153). Moreover, consistent with the results in Experiment 1, significant trial-to-trial negative correlations between trial number and saccade peak velocity were observed in the anti-saccade task, as a voluntary saccade task (Figure 6F, *t*(23) = 3.832, *p* < 0.001, d = 0.782), but not in the pro-saccade task (Figure 6E, *t*(23) = 1.843, *p* = 0.078, d = 0.376). Saccade amplitude was not modulated by time-on-task in both tasks (Figure 6G, all ps > 0.4). Trial-to-trial correlations between trial number and saccade amplitude were not significant in both tasks (Figure 6H: pro: *t*(23) = 0.33, *p* = 0.744, d = 0.067; Figure 6I: anti: *t*(23) = 1.414, *p* = 0.171, d = 0.289). As shown in Figure 6J, the main sequence slope was not significantly modulated by time-on-task, though there was a trend (F(2,46) = 0.986, *p* = 0.381, η*_p_*^2^ = 0.041), and other effects were not significant (all *p* > 0.1). These effects were significant on trial-to-trial correlational analysis in the pro-saccade task (pro: Figure 6K, *t*(23) = 2.304, *p* = 0.031, d = 0.47; anti: Figure 6L, *t*(23) = 0.412, *p* = 0.684, d = 0.084). Overall, similar to the results in Experiment 1, saccade peak velocities were reduced as time-on-task increased, particularly in the voluntary saccade task.

### 3.7. Tonic Pupil Size Effects on Saccade Latency and Metrics in the Pro- and Anti-Saccade Task

**Figure 7:**
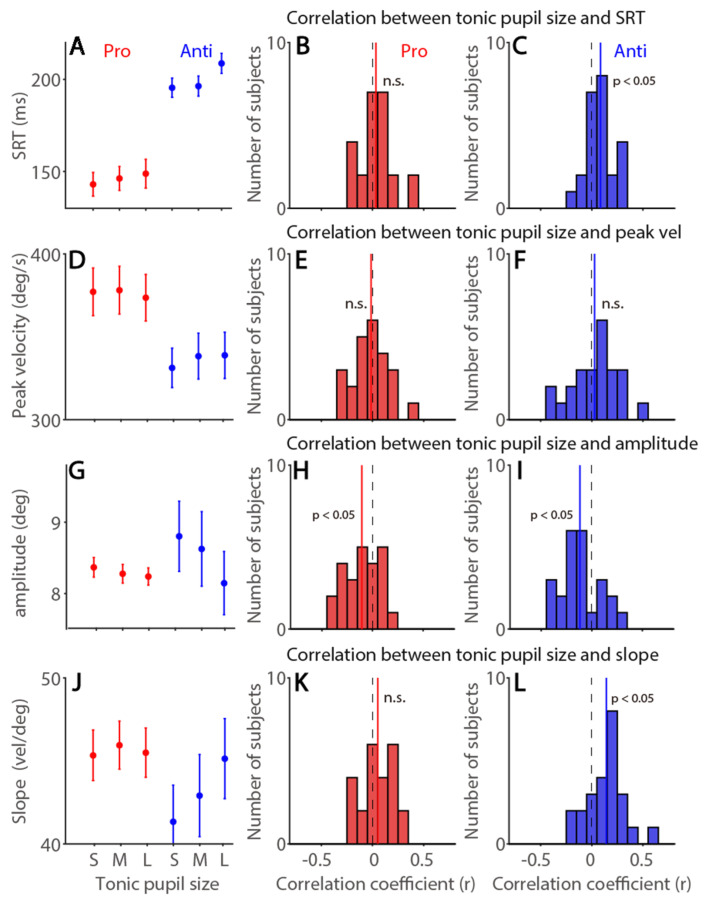
**Tonic pupil size effects on saccade latency and metrics in the pro- and anti-saccade task.** (**A**) Saccade reaction times (SRTs) at different tonic pupil size conditions. (**B**,**C**) Distribution of correlation coefficients for the relationship between tonic pupil size and SRT in the pro-saccade (**B**) and anti-saccade (**C**) task for all subjects (*n* = 24). (**D**) Saccade peak velocity at different tonic pupil size conditions. (**E**,**F**) Distribution of correlation coefficients for the relationship between tonic pupil size and saccade peak velocity in the pro-saccade (**E**) and anti-saccade (**F**) task for all subjects. (**G**) Saccade amplitude at different tonic pupil size conditions. (**H**,**I**) Distribution of correlation coefficients for the relationship between tonic pupil size and saccade amplitude in the pro-saccade (**H**) and anti-saccade (**I**) task for all subjects. (**J**) Main sequence slope (peak velocity/amplitude) at different tonic pupil size conditions. (**K**,**L**) Distribution of correlation coefficients for the relationship between tonic pupil size and slope in the pro-saccade (**K**) and anti-saccade (**L**) task for all subjects. The error bars represent ± standard error range (across participants). The vertical black dashed line represents a zero value of the correlation coefficient (r = 0), and the vertical colored line represents the mean value of the correlation coefficient. Pro: pro-saccade task. Anti: anti-saccade task. S: small, M: mid, L: large. SRT: saccade reaction times. n.s.: not significant.

We further examined tonic pupil size effects on saccade latency and dynamics in the pro- and anti-saccade task (tonic pupil size was defined as 0 to 100 ms after the instructed saccade cue onset). As shown in Figure 7A, correct SRTs were faster in the pro-saccade compared to the anti-saccade task (F(1,23) = 80.114, *p* < 0.001, η*_p_*^2^ = 0.777), and time-on-task effects were significant (time: F(2,46) = 6.108, *p* = 0.004, η*_p_*^2^ = 0.21; interaction: F(2,46) = 2.374, *p* = 0.104, η*_p_*^2^ = 0.094). Larger tonic pupil size correlated with longer SRTs in the anti-saccade task (Figure 7C, *t*(23) = 3.18, *p* = 0.004, d = 0.649) but not in the pro-saccade task (Figure 7B, *t*(23) = 1.026, *p* = 0.316, d = 0.209). There were higher saccade peak velocities in the pro-saccade task than in the anti-saccade task (Figure 7D, F(1,23) = 30.762, *p* < 0.001, η*_p_*^2^ = 0.572), and other effects were not significant (all *p* > 0.1). No trial-to-trial correlations were observed (pro: Figure 7E, *t*(23) = 0.412, *p* = 0.684, d = 0.084; anti: Figure 7F, *t*(23) = 0.582, *p* == 0.566, d = 0.119). Saccade amplitude was modulated by tonic pupil size (Figure 7G, time-on-task: F(2,46) = 5.657, *p* = 0.006, η*_p_*^2^ = 0.197; task: F(1,23) = 0.277, *p* = 0.604, η*_p_*^2^ = 0.012; interaction: F(2,46) = 3.33, *p* = 0.045, η*_p_*^2^ = 0.126). Trial-to-trial correlational analysis showed larger tonic pupil size correlating with smaller saccade amplitude (pro: Figure 7H, *t*(23) = 3.074, *p* = 0.005, d = 0.628; anti: Figure 7I, *t*(23) = 2.892, *p* = 0.008, d = 0.59). Main sequence slope was modulated by tonic pupil size (Figure 7J, time-on-task: F(2,46) = 6.767, *p* = 0.003, η*_p_*^2^ = 0.227; task: F(1,23) = 2.23, *p* = 0.149, η*_p_*^2^ = 0.088; interaction: F(2,46) = 6.084, *p* = 0.005, η*_p_*^2^ = 0.209), and trials with larger tonic pupil size correlated with steeper slope, particularly in the anti-saccade task (pro: Figure 7K, *t*(23) = 1.622, *p* = 0.118, d = 0.331; anti: Figure 7L, *t*(23) = 3.756, *p* = 0.001, d = 0.767). Again, we computed the partial correlation coefficient to control the effect of trial number (Supplementary Table S2 for summary). As shown in Supplementary Figure S4, the same patterns of the results were obtained in correlational analyses, again suggesting that tonic pupil size provides unique information to account for some fluctuations of saccade dynamics on a trial-to-trial basis. In summary, voluntary saccade responses were modulated by tonic pupil size, with increased main sequence slope and decreased saccade amplitude as tonic pupil size increased.

## 4. Discussion

To understand the effects of fatigue and arousal in trial-to-trial variability in humans performing cognitive saccade tasks, we examined time-on-task and tonic pupil size effects on saccade latency and dynamics. Time-on-task effects on a trial-to-trial basis were observed, with decreased voluntary saccade peak velocities, reduced tonic pupil size and phasic pupillary responses as time-on-task increased. We further found that tonic pupil size significantly affected saccade latency and dynamics, with reliably increased main sequence slope as tonic pupil size increased. Importantly, tonic pupil size effects were still significant when time-on-task effects were considered using partial correlation, implying that tonic pupil size provides unique signals accounting for trial-to-trial variability in human performance. Although phasic pupillary responses were also modulated by tonic pupil size, this modulation can be explained mostly by the law of initial values, so larger pupil dilation but smaller pupil constriction responses were obtained when tonic pupil size was smaller. In summary, saccade dynamics and tonic pupil size were sensitive to fatigue and arousal level, providing a valuable tool to examine human performance.

### 4.1. Time-On-Task Effects on Saccades and Pupil Size

Fatigue effects have been extensively studied to develop an effective model for automated fatigue detection [9,10,13]. As fatigue level should increase over time in humans performing a task (time-on-task effects), time-on-task should be observed in saccade dynamics. Consistent with previous research [11,12,13,15], saccade peak velocities decreased as time-on-task increased, supporting the idea that saccade peak velocity is sensitive to arousal level [16]. We further found that these time-on-task effects on saccade peak velocity were particularly pronounced in the voluntary saccade task (memory-guided saccade and anti-saccade task), suggesting voluntary saccade peak velocity to be an effective index of fatigue level. In addition, because tonic pupil size and phasic pupillary responses are thought to reveal different neural processes [41,66,67] and correlate with peripheral indices [40], we also examined time-on-task effects on these pupillary responses. Tonic pupil size, as an arousal index, should correspondingly decrease over time because fatigue level should increase over time. Consistently, we found that tonic pupil size decreased as trial number increased. More importantly, we found that phasic pupillary responses evoked by a bright patch stimulus (pupil light reflex) or an emotional sound stimulus (pupil dilation) both decreased as trial number increased, together showing similar time-on-task effects on saccade and pupillary responses, arguably through overlapping neural pathways. Because participants were not asked to report their fatigue level in each trial, future research that incorporates a trial-to-trial subjective report of fatigue level is needed to examine the relationship between these indices and subjective fatigue level.

### 4.2. Tonic Pupil Size Effects in Saccade and Phasic Pupillary Responses

Pupil size is modulated by various arousal-related factors, including not only fatigue level but also emotional arousal and valence [23,24,25,26,27,28,29], providing an index for the general arousal level. To directly investigate the relationship between tonic pupil size and other measurements, we correlated tonic pupil size with saccade and pupil responses on a trial-to-trial basis. Trial-to-trial correlations between tonic pupil size and saccade slope were reliably observed across two voluntary saccade tasks; in particular, larger tonic pupil sizes correlated with steeper main sequence slope. These results suggested that main sequence slope in voluntary saccades was effective to index arousal level, and higher arousal level correlated with higher saccade peak velocities when saccade size was controlled. More importantly, these correlations were still pronounced even when time-on-task was considered using partial correlation analysis. These results suggest that tonic pupil size provides unique signals that can account for trial-to-trial variability in human performance. Our results were generally consistent with the literature, showing arousal effects on saccade behavior in humans performing a range of tasks [16,70,71,72,73,74,75]. Moreover, significant correlations between tonic pupil size and phasic pupillary responses were obtained, with larger tonic pupil size correlating with larger pupil constriction size but with smaller pupil dilation size. These effects can be explained by the law of initial values [68]; that is, there is more room for the pupil to constrict when tonic pupil size is larger, whereas there is more room for the pupil to dilate when tonic pupil size is smaller. These results suggest that caution is needed to use phasic pupillary responses as an arousal index because different effects could be obtained between pupil constriction and dilation responses.

### 4.3. Neural Mechanisms for Coordinated Saccades and Phasic Pupillary Responses

What are the neural mechanisms underlying these fatigue and arousal effects between saccades and pupil size? The SC is a subcortical structure that is known for its role in saccade and attention control [76,77]. The SC receives important control signals from a range of cortical and subcortical structures, for example, multisensory signals from the retina and inferior colliculus, cognitive signals from the FEF, and arousal signals from the LC [78,79,80,81], and projects to the premotor circuitry in the brainstem reticular formation and the spinal cord to initiate movements of the eyes and head [82,83]. The role of the SC has been extended to pupil size [30,35,37]. Anatomically, the SC connects the Edinger-Westphal nucleus (EW) in the pupil control pathway mostly indirectly via central mesencephalic reticular formation [84,85], and the EW nucleus projects to the ciliary ganglion with both excitatory and inhibitory connections [86], together providing the necessary connections to change pupil size. Pupil dilation is evoked by SC (or FEF) microstimulation without evoking saccades [30,31,32,36]. Moreover, saccades and pupil responses evoked by suprathreshold microstimulation of the SC correlated [31], and the correlation between saccades and pupil responses is disrupted with continuous theta-burst stimulation over the FEF in humans [38]. Furthermore, pupil-linked arousal level modulates saccade responses evoked by SC microstimulation [87]. Together, these suggest that the pathway mediated by the SC receives arousal-related signals, arguably from the LC and other areas, to coordinate similar effects observed between saccades and pupil size. Further investigation with direct neuronal recordings in behaving animals is required to understand neural correlates of observed coordination between saccades and pupil size.

## 5. Conclusions

Modern eye trackers that measure eye position and pupil size with high spatial and temporal resolution provide a powerful tool for understanding human performance. In contrast to most research that focuses on single response, here, we demonstrated fatigue and arousal influenced both saccade and pupillary responses, and we suggest a role of the SC in such behavior. In addition, fatigue and arousal effects were particularly pronounced in voluntary saccades, suggesting the importance of developing algorithms to classify voluntary saccades for automatic fatigue detection. Saccades and pupil size are frequently used to infer various brain functions in health and disease [4,88]. By showing trial-to-trial correlations between tonic pupil size and saccade dynamics, our findings provide a potential source of behavioral biomarkers related to oculomotor function for diseases. In conclusion, saccade and pupillary responses provide valuable information for cognitive and neural processing. It is thus important to simultaneously examine these indices to understand human behavior.

## Figures and Tables

**Table 1:** Summary for the mean correlation coefficient values for each correlational analysis in Experiment 1 (*n* = 28). * indicates statistically significant. Numbers in the bracket indicates the percentage of participants showing the same sign of correlation. SRT: saccade reaction time. Peak Vel: saccade peak velocity. Amplitude: saccade amplitude. Slope: the main sequence slope (peak velocity/amplitude). Tonic pu: tonic pupil size. Phasic pu: phasic pupil constriction. Visual: visually guided saccade task. Memory: memory-guided saccade task.

Exp. 1		SRT	Peak Vel	Amplitude	Slope	Tonic pu	Phasic pu
**Visual**	**Trial number**	−0.12 * (71%)	−0.06 (68%)	−0.01 (46%)	−0.04 (68%)	−0.36 * (89%)	−0.15 * (75%)
**Memory**		−0.06 * (71%)	−0.12 * (79%)	−0.05 * (61%)	−0.05 (57%)	−0.37 * (86%)	−0.16 * (68%)
**Visual**	**Tonic pupil size**	−0.02 (57%)	0.11 * (82%)	−0.03 (68%)	0.14 * (82%)		−0.65 * (93%)
**Memory**		0.01 (54%)	0.18 * (89%)	0.01 (47%)	0.16 * (79%)		−0.65 * (93%)

**Table 2:** Summary for the mean correlation coefficient values for each correlational analysis in Experiment 2 (*n* = 24). * indicates statistically significant. Numbers in brackets indicate the percentage of participants showing the same sign of correlation. SRT: saccade reaction time. Peak Vel: saccade peak velocity. Amplitude: saccade amplitude. Slope: the main sequence slope (peak velocity/amplitude). Tonic pu: tonic pupil size. Phasic pu: phasic pupil dilation. Pro: pro-saccade task. Anti: anti-saccade task.

Exp. 2		SRT	Peak Vel	Amplitude	Slope	Tonic pu	Phasic pu
**Pro**	**Trial number**	−0.07 (63%)	−0.09 (67%)	0.02 (54%)	−0.08 * (63%)	−0.18 * (71%)	−0.08 * (79%)
**Anti**		−0.06 (54%)	−0.19 * (75%)	−0.08 (71%)	−0.02 (54%)		
**Pro**	**Tonic pupil size**	0.03 (50%)	−0.01 (63%)	−0.11 * (71%)	0.05 (62%)		−0.45 * (100%)
**Anti**		0.09 * (79%)	−0.03 (58%)	−0.12 * (75%)	0.14 * (75%)		

## Data Availability

Data are available from the authors upon reasonable request following the publication.

## Supplementary Materials

The following supporting information can be downloaded at: https://www.mdpi.com/article/10.3390/ijerph19159234/s1, Figure S1: Partial correlation between tonic pupil size and phasic pupil constriction with trial number controlled in Experiment 1. Figure S2: Partial correlation between tonic pupil size and saccade latency and metrics with trial number controlled in Experiment 1. Figure S3: Partial correlation between tonic pupil size and phasic pupil dilation with trial number controlled in Experiment 2. Figure S4: Partial correlation between tonic pupil size and saccade latency and metrics with trial number controlled in Experiment 2. Table S1: Summary for the mean partial correlation coefficient values for each correlational analysis in Experiment 1 (N = 28). Table S2: Summary for the mean partial correlation coefficient values for each correlational analysis in Experiment 2 (N = 24).
