# Supplementary material for: Fatigue and Arousal Modulations Revealed by Saccade and Pupil Dynamics
Source: Int J Environ Res Public Health. 2022 Jul 28;19(15):9234. doi: 10.3390/ijerph19159234 (PMC9367726; doi:10.3390/ijerph19159234)
Supplement: Supplementary file 1 [file ijerph-19-09234-s001.zip › ijerph-1774592-supplementary.pdf]

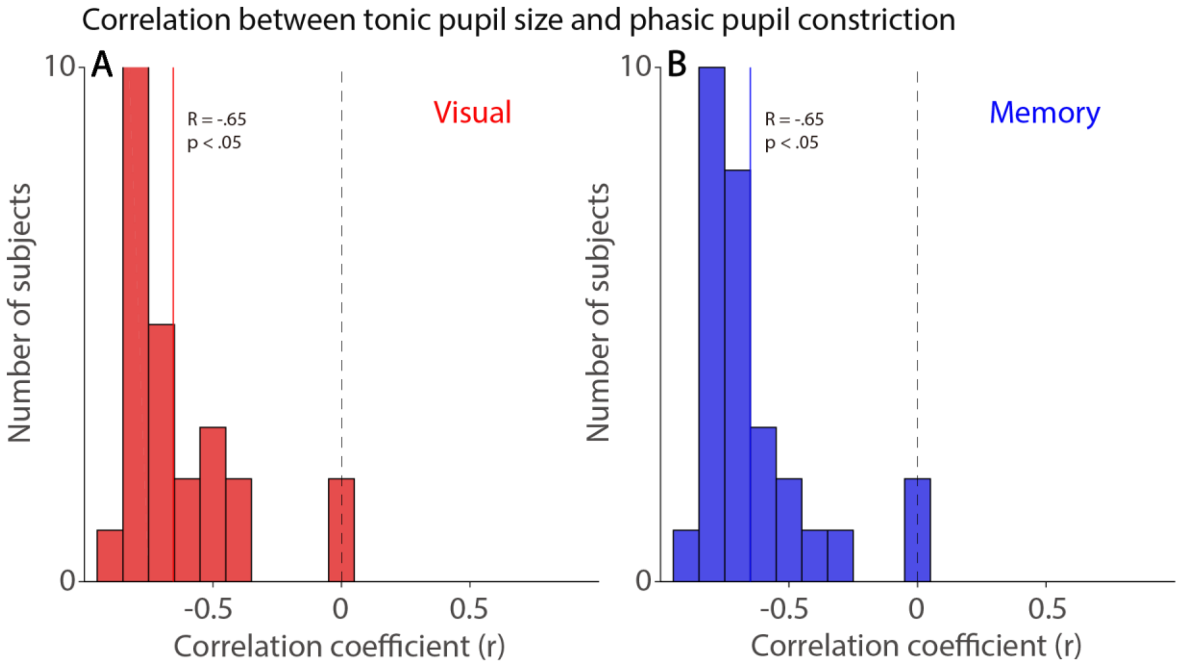

Supplementary Figure S1. Partial correlation between tonic pupil size and phasic pupil constriction with trial number controlled in Experiment 1. (A,B) Distribution of correlation coefficients for the relationship between tonic pupil size and phasic pupil constriction size in the visually-guided (A) and memory-guided (B) saccade task for all subjects. The vertical black dashed line represents a zero value of the correlation coefficient ($r = 0$), and the vertical colored line represents the mean value of the correlation coefficient. Visual: visually-guided saccade task. Memory: memory-guided saccade task.

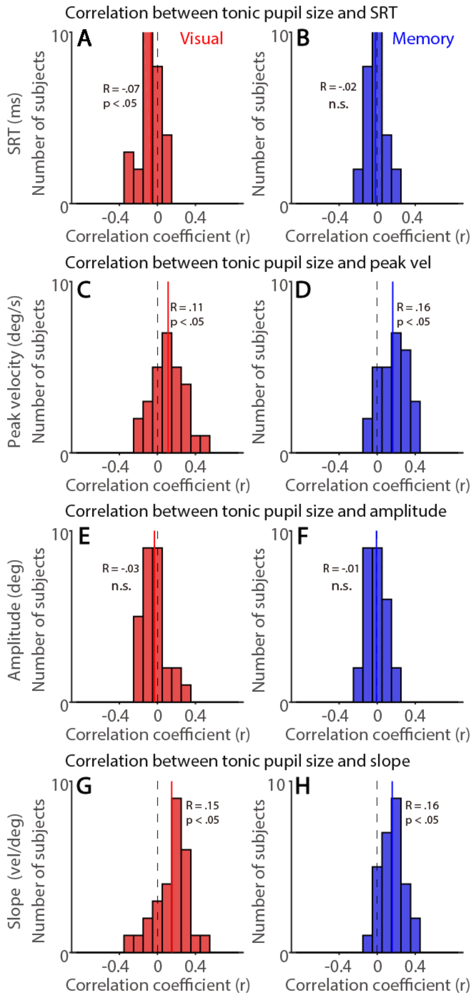

Supplementary Figure S2. Partial correlation between tonic pupil size and saccade latency and metrics with trial number controlled in Experiment 1. (A,B) Distribution of correlation coefficients for the relationship between tonic pupil size and SRT in the visually-guided (A) and memory-guided (B) saccade task for all subjects. (C,D) Distribution of correlation coefficients for the relationship between tonic pupil size and saccade peak velocity in the visually-guided (C) and memory-guided (D) saccade task for all subjects. (E,F) Distribution of correlation coefficients for the relationship between tonic pupil size and saccade amplitude in the visually-guided (E) and memory-guided (F) saccade task for all subjects. (G,H) Distribution of correlation coefficients for the relationship between tonic pupil size and main sequence slope (peak velocity/amplitude) in the visually-guided (G) and memory-guided (H) saccade task for all subjects. The vertical black dashed line represents a zero value of the correlation coefficient (r = 0), and the vertical colored line represents the mean value of the correlation coefficient. Visual: visually-guided saccade task. Memory: memory-guided saccade task. n.s.: not significant.

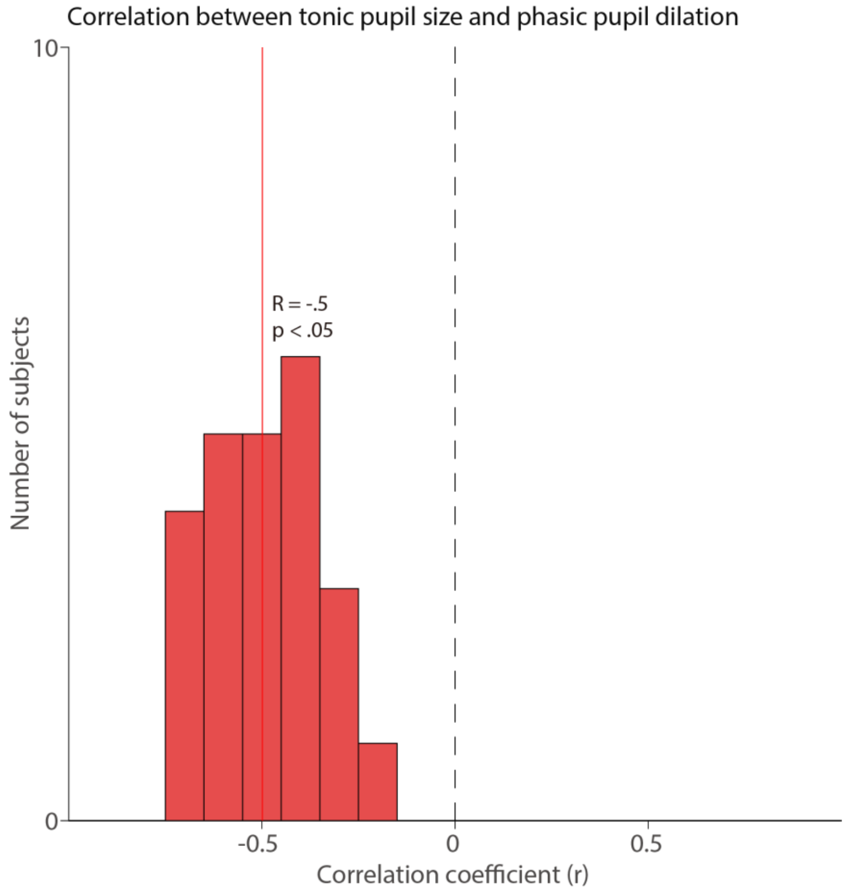

Supplementary Figure S3. Partial correlation between tonic pupil size and phasic pupil dilation with trial number controlled in Experiment 2. (A,B) Distribution of correlation coefficients for the relationship between tonic pupil size and phasic pupil constriction size in the visually-guided (A) and memory-guided (B) saccade task for all subjects. The vertical black dashed line represents a zero value of the correlation coefficient (r = 0), and the vertical colored line represents the mean value of the correlation coefficient.

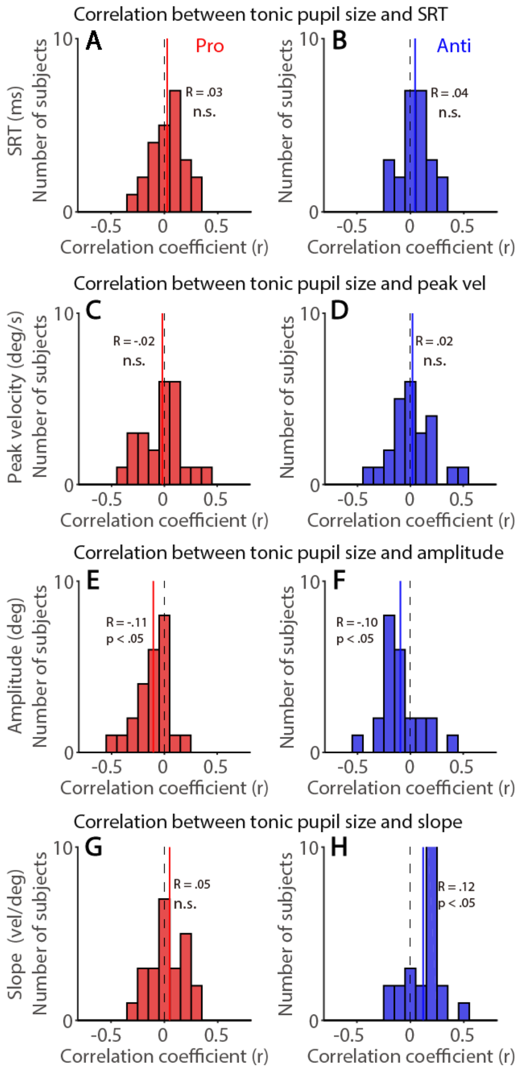

Supplementary Figure S4. Partial correlation between tonic pupil size and saccade latency and metrics with trial number controlled in Experiment 2. (A,B) Distribution of correlation coefficients for the relationship between tonic pupil size and SRT in the visually-guided (A) and memory-guided (B) saccade task for all subjects. (C,D) Distribution of correlation coefficients for the relationship between tonic pupil size and saccade peak velocity in the visually-guided (C) and memory-guided (D) saccade task for all subjects. (E,F) Distribution of correlation coefficients for the relationship between tonic pupil size and saccade amplitude in the visually-guided (E) and memory-guided (F) saccade task for all subjects. (G,H) Distribution of correlation coefficients for the relationship between tonic pupil size and main sequence slope (peak velocity/amplitude) in the visually-guided (G) and memory-guided (H) saccade task for all subjects. The vertical black dashed line represents a zero value of the correlation coefficient (r =

0), and the vertical colored line represents the mean value of the correlation coefficient. Pro: pro-saccade task. Anti: anti-saccade task. n.s.: not significant.

Table S1: Summary for the mean partial correlation coefficient values for each correlational analysis in Experiment 1 (N = 28). * indicates statistically significant. Numbers in the bracket indicates the percentage of participants showing the same sign of correlation. SRT: saccade reaction time. Peak Vel: saccade peak velocity. Amplitude: saccade amplitude. Slope: the main sequence slope (peak velocity/amplitude). Tonic pu: tonic pupil size. Phasic pu: phasic pupil constriction. Visual: visually-guided saccade task. Memory: memory-guided saccade task.

Exp 1		SRT	Peak Vel	Amplitude	Slope	Phasic pu
**Visual**	**Tonic pupil size**	-0.07* (71%)	0.11* (75%)	-0.03 (61%)	0.15* (75%)	-0.65* (93%)
**Memory**		-0.02 (61%)	0.16* (79%)	-0.01 (61%)	0.16* (82%)	-0.65* (96%)

Table S2: Summary for the mean partial correlation coefficient values for each correlational analysis in Experiment 2 (N = 24). * indicates statistically significant. Numbers in the bracket indicates the percentage of participants showing the same sign of correlation. SRT: saccade reaction time. Peak Vel: saccade peak velocity. Amplitude: saccade amplitude. Slope: the main sequence slope (peak velocity/amplitude). Tonic pu: tonic pupil size. Phasic pu: phasic pupil dilation. Pro: pro-saccade task. Anti: anti-saccade task.

Exp 2		SRT	Peak Vel	Amplitude	Slope	Phasic pu
**Pro**	**Tonic pupil size**	0.03 (50%)	-0.02 (50%)	-0.11* (71%)	0.05 (67%)	-0.50* (100%)
**Anti**		0.04 (71%)	0.02 (50%)	-0.10* (75%)	0.12* (79%)	
